# Purple Pleural Effusion due to *Acinetobacter baumannii* Infection: A Rare Case Report

**DOI:** 10.1155/carm/6904355

**Published:** 2025-10-06

**Authors:** Sara Heidari, Forough Kalantari, Elham Kalantari

**Affiliations:** ^1^Department of Medicine, School of Medicine, Isfahan University of Medical Sciences, Isfahan, Iran; ^2^Department of Nuclear Medicine, Rasoul Akram Hospital, Iran University of Medical Sciences, Tehran, Iran; ^3^Department of Pulmonology, School of Medicine, Isfahan University of Medical Sciences, Isfahan, Iran

**Keywords:** *Acinetobacter baumannii*, bacterial infection, purple pleural effusion

## Abstract

**Background:**

Pleural effusion (PE) is a frequent clinical condition with diverse etiologies including heart failure, infections, and malignancies. While the color of pleural fluid is rarely considered diagnostic, unusual discolorations may offer important clinical clues. We report what appears to be only the third documented case of purple PE (PPE) in the medical literature. This case was associated with *Acinetobacter baumannii* infection, a rarely reported cause of PPE, providing additional insight into its potential pathophysiology.

**Case Presentation:**

A 54 year-old obese male with multiple comorbidities—including COPD, heart failure, and recent pulmonary embolism—was admitted with acute respiratory failure. He was found to have a right-sided PE requiring drainage. Initial fluid analysis revealed an exudative, lymphocyte-predominant effusion with no evidence of infection. However, the fluid in the drainage bag gradually turned deep purple, and *Acinetobacter baumannii* was later isolated from the catheter and collection bag. The patient was treated with colistin and meropenem, with full clinical recovery and resolution of discoloration.

**Discussion:**

Although the exact mechanism remains unclear, PPE may share pathophysiologic pathways with Purple Urine Bag Syndrome, involving bacterial metabolism of tryptophan-derived compounds. This case highlights the importance of visual inspection of pleural fluid and emphasizes that unusual discoloration—while not diagnostic in itself—should prompt thorough microbiological and biochemical evaluation.

**Key Clinical Message:**

Unusual pleural fluid discoloration should prompt immediate microbiological evaluation, as it may indicate infection with uncommon or multidrug-resistant organisms, even in the absence of typical infection signs.

## 1. Introduction

Pleural effusion (PE), the most common pleural disease, is characterized by excess fluid accumulation within the pleural spaces. This condition is a consequence of an imbalance between pleural fluid formation and absorption [1, 2]. PE can result from various underlying conditions, such as left-sided heart failure, liver cirrhosis, pneumonia, tuberculosis, pulmonary embolism, or malignancies. While chest imaging is used to diagnose PE and assess its extent, thoracentesis is necessary to identify the underlying cause, thereby tailoring more effective treatment interventions. Unusual pleural fluid discolorations—such as green in chronic empyema, black in Aspergillus infection, or brown in amebic pleural effusion—are rare but can provide important diagnostic clues. Purple pleural effusion (PPE) is exceptionally rare, with only two previous cases reported in the literature.

To the best of our knowledge, only two prior cases of PPE have been reported in the literature. The first involved a Staphylococcus epidermidis infection following pacemaker insertion [3], and the second, published in 2023, was attributed to *Acinetobacter baumannii* in a diabetic patient with chronic kidney disease (CKD) [4]. Our case, the third of its kind, occurred in a patient with markedly different clinical features—morbid obesity, recent pulmonary embolism, and preserved renal function—and may thus offer new insights into the clinical spectrum and pathogenesis of PPE. Differential diagnoses for colored pleural effusions include hemorrhagic effusion, pigmented infections, malignancies with hemosiderin-laden cells, and infection with chromogenic bacteria such as Serratia marcescens.

Here, we present a rare case in which the drained pleural fluid was observed to be purple.

## 2. Case Presentation

A 54-year-old male patient, who was morbidly obese, presented to the emergency department complaining of progressive dyspnea, fatigue, retrosternal chest pain radiating to his left arm, and bilateral lower extremity edema. His symptoms were consistent with NYHA functional class III-IV. The patient had a recent history of 24-day hospitalization due to pulmonary thromboembolism (PTE) and was discharged one day before his current admission.

The patients had several preexisting comorbidities, including hypertension, heart failure, chronic obstructive pulmonary disease (COPD), deep vein thrombosis (DVT), obstructive sleep apnea, and fatty liver disease. Moreover, he was an opioid user and a former smoker.

During physical examination, the patient was somehow drowsy but oriented to time, place, and person. His vital signs were as follows: respiratory rate of 30 breaths per minute, heart rate of 120 beats per minute, blood pressure of 130/90 mmHg, axillary temperature of 36.5 degrees Celsius, and O_2_ saturation of 65% on room air, which improved to 93% after supplemental oxygen was provided. Lung auscultation revealed decreased breath sounds in the right hemithorax, and there was 3+ edema evident in the lower extremities. No other significant abnormalities were noted during the physical examination.

At initial evaluation, blood gas analysis was as follows: pH 7.31 (7.35–7.45), PaCO_2_ 80.6 mmHg (35–45), HCO_3_^−^^−^ 39.5 mmol/L (22–26), and PaO_2_ 82.5 mmHg (75–100). Noninvasive ventilation was started with an inspiratory pressure of 18 cmH_2_O, expiratory pressure of 6 cmH_2_O, and a backup respiratory rate of 13 breaths per minute to treat hypercapnic respiratory failure and improve oxygenation. Laboratory tests, including liver and kidney function tests, complete blood count (CBC), and electrolytes, were conducted, and all results were within reference ranges. The electrocardiogram (ECG) revealed no abnormalities. To assess pulmonary function, a spiral lung computed tomography (CT) scan of the chest was performed, revealing a moderate volume of right-sided pleural effusion. In the next step, an ultrasound-guided diagnostic therapeutic thoracocentesis was conducted to remove pleural effusion, yielding an exudative fluid with a serosanguinous appearance. The fluid analysis provided the following characteristics: pleural fluid WBC 200/μL (N 0–500), neutrophils 12%, lymphocytes 88%, protein 2.4 g/dL (serum 6.7 g/dL), LDH 669 U/L (serum 192 U/L; lab ULN 250 U/L), glucose 72 mg/dL, pH 7.20 (exudate by Light's criteria).

**Figure 1:**
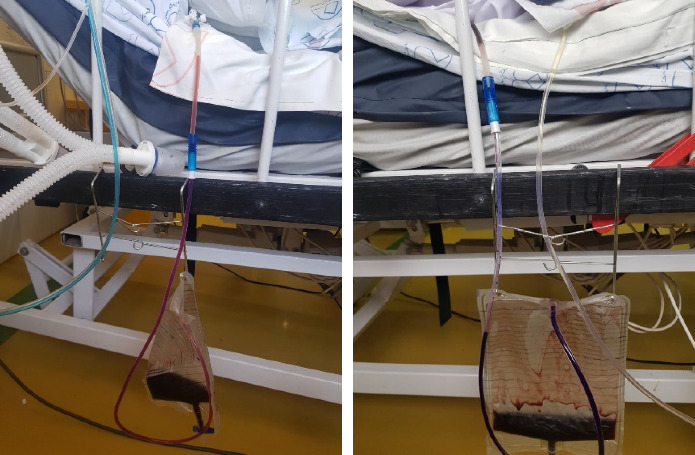
Deep purple discoloration noted within the chest-drain catheter and collection bag on Day 5, which resolved after targeted antibiotic therapy.

Pleural fluid pH was 7.2, and cytologic studies did not reveal any malignant cells. In view of the residual effusion volume following the initial thoracentesis, the ongoing dyspnea, and the pleural fluid analysis results, the decision was made to insert a catheter. Several days later, the drained fluid in the collection bag developed a deep purple discoloration (Figure 1), which prompted repeat cultures from both the catheter and the drainage bag. Initial pleural fluid samples obtained at thoracentesis were sterile with no organisms detected on Gram stain or culture. However, repeat samples collected from the drainage catheter and collection bag—following the appearance of purple discoloration—yielded *Acinetobacter baumannii*. The catheter and the fluid collected in the drainage bag were visibly discolored, appearing purple (Figure 1). Although the initial pleural fluid culture was negative, a repeat culture was obtained due to clinical deterioration and the unusual purple discoloration of the drained fluid. This decision was based on the visual change alone, anticipating the possibility of infection regardless of the initially negative culture result.

Microbial culture of specimens collected from the catheter and drainage bag confirmed the infection with *Acinetobacter baumannii*. Because discoloration was first noted within the drainage system, colonization or secondary infection of the catheter/bag was considered. Nonetheless, the patient's clinical response to targeted antibiotics and resolution of discoloration supported a clinically significant device-associated infection contributing to the presentation.

Due to pathogen's resistance to all treatments evaluated in the antimicrobial susceptibility test, microbial therapy was initiated with colistin (4.5 MU q12 h) and meropenem (2 g q8h). Given multidrug resistance and device-associated colonization/infection, combination therapy with colistin and meropenem was selected per local susceptibility and stewardship guidance. Following the administration of these antibiotics, the patient's condition significantly improved, and the purple discoloration was resolved.

During hospitalization, the patient developed rising serum creatinine levels, likely secondary to colistin-induced nephrotoxicity. As a result, the colistin dose was adjusted, and nephroprotective therapy was initiated with N-acetylcysteine (600 mg twice daily) and vitamin E (400 IU once daily). Over the following days, the patient's renal function gradually improved, with a corresponding decline in serum creatinine levels.

The patient reported significant relief from dyspnea and general fatigue. Follow-up chest imaging showed significant resolution of the effusion, and inflammatory markers (CRP and WBC count) normalized. Despite initial improvement in clinical symptoms and resolution of purple discoloration following antimicrobial therapy, residual loculated pleural effusion was noted, limiting complete lung expansion. Surgical consultation was obtained, and the patient subsequently underwent decortication to remove the fibrous pleural peel and restore full lung function. Postoperative recovery was uneventful, and the patient was discharged in stable condition with significant improvement in respiratory symptoms. A detailed chronological summary of the patient's clinical course is provided in Table 1.

## 3. Discussion

PPE is an uncommon medical condition in which plural fluid turns purple, typically due to bacterial infections. To the best of our knowledge, only two cases of PPF have been reported in the current medical literature. The first case involved a 60-year-old female patient with hypertension who developed a massive pneumothorax following pacemaker insertion in 2021 [3]. The second case was a young diabetic male with CKD, who developed a massive pleural effusion in 2023 [4]. In both cases, further investigations revealed bacterial infections of pleural fluid caused by *Staphylococcus epidermidis* in the first case and *Acinetobacter baumannii* in the latter.

Mehrotra et al. [3] described a 60-year-old hypertensive female who developed PPE following pacemaker insertion, in whom Staphylococcus epidermidis was isolated from pleural fluid. Agarwal et al. [4] later reported a young diabetic male with CKD who developed massive PPE due to *Acinetobacter baumannii*. Both patients improved with targeted antibiotic therapy. Compared with these cases, our patient had markedly different comorbidities, including morbid obesity, COPD, and recent pulmonary embolism, and A. baumannii was isolated from the drainage catheter. Interestingly, in our case, the discoloration of pleural fluid became apparent only after some time, which led to repeat cultures despite an initially negative result. These differences broaden the clinical spectrum of PPE and suggest that device-associated infection may also represent an additional explanation.

Differential diagnosis of discolored pleural fluid:

• Hemorrhagic/Red: hemothorax, malignant pleural effusion (RBCs, high hematocrit) [5].
• Green: bilothorax (biliopleural fistula) or *Pseudomonas* empyema (pyocyanin/pyoverdine) [6].
• Black: *Aspergillus niger* infection, metastatic melanoma, long-standing hemothorax [7].
• Brown: amebic empyema or old blood [8].
• Purple: rare; reported with S. epidermidis and A. baumannii; mechanism likely bacterial metabolism of indoxyl derivatives (sulfatase/phosphatase).

Although the color of pleural fluid is not itself diagnostic, it may provide an important early visual clue to underlying pathology. Recognition of unusual discoloration can alert clinicians to the possibility of infection or atypical disease processes, even in the absence of classical clinical or laboratory features. In our case, the observation of purple discoloration directly prompted repeat microbiological cultures, which identified *Acinetobacter baumannii* and guided appropriate antimicrobial therapy. Thus, unusual pleural fluid color should be considered a practical clinical signal that warrants thorough microbiological and biochemical investigation to avoid delays in diagnosis and treatment.

Generally, medical diseases and conditions associated with the purple coloration of body fluids are rare. Purple Urine Bag Syndrome (PUBS) and PPE are prominent examples of such conditions. In PUBS, which is manifested mainly in individuals with indwelling urinary catheters, gut bacteria metabolize dietary tryptophan into indole, which then enters the bloodstream. In the liver, indole converts to indoxyl sulfate (IS), excreted into the urine. This compound is further metabolized by gram-negative bacterial enzymes, including sulfatases and phosphatases, resulting in a mixture of indigo, a blue substance, and indirubin, a red compound, which together cause purple coloration. While the underlying mechanisms of PPE are not fully understood, analogies with PUBS suggest that bacterial metabolism of tryptophan-derived compounds—particularly through enzymes such as sulfatases and phosphatases—may lead to pigment formation. In our case, *Acinetobacter baumannii* was identified from the drainage catheter and collection bag, but not from initial pleural fluid samples, raising the possibility of secondary colonization, contamination, or a delayed superinfection. Moreover, this organism is not classically associated with indole metabolism, and its role in pigment production remains uncertain. The possibility of undetected coinfection with indole-producing gram-negative bacteria (such as E. coli, Proteus, or Klebsiella) cannot be fully excluded.  Table 2 compares the present case with the two previously published PPE cases, highlighting differences in comorbidities, causative organisms, and clinical outcomes.

The pleural fluid in this case was lymphocyte-predominant (88%), which is unusual in acute bacterial infections that typically show neutrophilic predominance. Lymphocyte predominance can be seen in subacute/parapneumonic states, malignancy, tuberculosis, and chronic inflammatory conditions. In our patient, timing of sampling, prior hospitalization, and device-associated infection may explain a non-neutrophilic profile despite bacterial growth from the drainage system. Repeat cytology and microbiology remain essential when clinical trajectory is discordant with initial results. This cytologic profile, along with the timing of culture positivity and the patient's comorbidities (e.g., heart failure, COPD, and obesity), suggests a more complex or delayed immunologic response and highlights the need for cautious interpretation of findings.

Interestingly, the microorganisms responsible for the purple discoloration of urine in PUBS are primarily *Escherichia coli*, *Proteus mirabilis*, *Klebsiella pneumoniae*, Enterococcus, and *Pseudomonas aeruginosa*, with only 0.6% of cases involving *Acinetobacter baumannii* [9]. However, the latest documented PPE case, reported in 2023, and our case of PPE have been linked to *Acinetobacter baumannii* infection. This suggests that while the general mechanisms may be similar, the specific microbial agents can vary.

While bacterial enzymes are involved in the development of purple discoloration in both pleural fluid and urine, it is important to consider other factors that may contribute to these conditions. For example, factors increasing IS serum levels, such as impaired renal function, dietary habits, and the gut microbiome, may play a role in developing these conditions. CKD and acute kidney injury can compromise renal function. A high-protein diet can increase tryptophan availability, producing more IS. Moreover, fiber, a nondigestible carbohydrate in the intestine, can bind to IS, reducing its absorption into the bloodstream. Consequently, a low-fiber diet can result in a higher serum level of IS. These host and dietary factors may modulate substrate availability (IS), potentially amplifying pigment formation in susceptible infections [10, 11].

In conclusion, it is thought that bacterial enzymes play a significant role in the development of purple discoloration observed in both pleural fluid and urine. It is important to note that patients with PPE may not necessarily display typical signs of infection, such as fever or shock, which may result in delayed diagnosis. Therefore, while purple discoloration should prompt thorough microbiological and clinical evaluation, antibiotic therapy should be guided primarily by the presence of clinical signs of infection and laboratory evidence—not solely by color [9, 12].

This case expands the clinical spectrum of PPE and highlights the importance of integrating visual, microbiologic, and clinical data in managing such rare presentations.

## 4. Conclusion

PPE is an exceptional visual clue that should not be dismissed as incidental. When encountered, clinicians should obtain repeat cultures—explicitly including catheter and collection-bag samples—and consider device-associated infection by uncommon or multidrug-resistant organisms such as A. baumannii. Management should be guided by clinical status and microbiology rather than color alone. Early recognition and targeted therapy can prevent delays and improve outcomes.

## Figures and Tables

**Table 1:** Chronological summary of the patient's clinical course.

Hospital day	Clinical events and interventions	Key findings/notes
−24 to −1	Previous hospitalization for pulmonary thromboembolism (24 days); discharged 1 day before current admission	Stable at discharge
0	Readmission with acute respiratory failure; noninvasive ventilation initiated	RR 30/min, HR 120 bpm, BP 130/90 mmHg, SpO_2_ 65% ⟶ 93% with O_2_; moderate right-sided pleural effusion on CT
1	Ultrasound-guided thoracentesis performed	Serosanguinous exudate, lymphocyte-predominant; initial culture negative
2–4	Considering the residual pleural fluid volume and analysis and ongoing dyspnea	Chest tube inserted for continuous drainage and repeat sampling
5	Deep purple discoloration first noted in drainage system	Prompted repeat cultures from catheter and drainage bag
7	Cultures from catheter and bag positive for *Acinetobacter baumannii*	Antimicrobial therapy initiated colistin 4.5 MU q12 h + meropenem 2 g q8h
7–15	Continued targeted antimicrobial therapy	Gradual clinical improvement; purple discoloration resolved
13	Significant radiologic improvement	Residual loculated effusion noted
14	Residual loculated pleural effusion	Surgical consultation obtained
15	Discharged in stable condition; planned surgical management	
Postdischarge	Surgical decortication performed to remove fibrous pleural peel	Full lung expansion achieved; uneventful recovery

**Table 2:** Summary of reported cases of purple pleural effusion (PPE) in the literature.

Source	Age/sex	Comorbidity	Pathogen	Culture source	Outcome
Mehrotra et al., 2022 [3] (India)	60/F	Postpacemaker	*S. epidermidis*	Pleural fluid	Recovery
Agarwal et al., 2023 [4] (BMJ)	∼30 s/M	DM, CKD	*A. baumannii*	Pleural fluid	Recovery
Present case	54/M	Obesity, HF, COPD, recent PTE	*A. baumannii*	Catheter/bag (initial pleural fluid sterile)	Recovery; decortication

*Note:* Summary of clinical features, comorbidities, identified pathogens, culture sources, and outcomes of all reported cases of purple pleural effusion, including the present case. PPE remains extremely rare, with only two prior reports before this study.
